# Reusable three-dimensional nanostructured substrates for surface-enhanced Raman scattering

**DOI:** 10.1186/1556-276X-9-25

**Published:** 2014-01-13

**Authors:** Zhendong Zhu, Qunqing Li, Benfeng Bai, Shoushan Fan

**Affiliations:** 1 Department of Physics & Tsinghua-Foxconn Nanotechnology Research Center, State Key Laboratory of Low-Dimensional Quantum Physics, Tsinghua University, Beijing 100084, China; 2 Collaborative Innovation Center of Quantum Matter, Beijing, China; 3 Department of Precision Instruments, State Key Laboratory of Precision Measurement Technology and Instruments, Tsinghua University, Beijing, China

**Keywords:** Three-dimensional (3D) nanostructure, Nanosphere lithography, Surface-enhanced Raman scattering (SERS)

## Abstract

To date, fabricating three-dimensional (3D) nanostructured substrate with small nanogap was a laborious challenge by conventional fabrication techniques. In this article, we address a simple, low-cost, large-area, and spatially controllable method to fabricate 3D nanostructures, involving hemisphere, hemiellipsoid, and pyramidal pits based on nanosphere lithography (NSL). These 3D nanostructures were used as surface-enhanced Raman scattering (SERS) substrates of single Rhodamine 6G (R6G) molecule. The average SERS enhancement factor achieved up to 10^11^. The inevitably negative influence of the adhesion-promoting intermediate layer of Cr or Ti was resolved by using such kind of 3D nanostructures. The nanostructured quartz substrate is a free platform as a SERS substrate and is nondestructive when altering with different metal films and is recyclable, which avoids the laborious and complicated fabricating procedures.

## Background

Raman spectroscopy is a powerful and label-free tool for identifying molecular species because the signals of re-emitted Raman photons address for all molecular species and correspond to a particular set of vibration modes. However, the Raman signal is very weak because Raman scattering is an inelastic scattering process of photon, only one in every 10^7^ photon incidence on a molecule undergoing Raman scattering, and it has a second-order dipole transition nature. Fortunately, it was discovered that the signals of Raman scattering could be amplified enormously by molecules contacting with a textured or patterned special noble metal surface, termed as surface-enhanced Raman scattering (SERS) [1,2]. Commonly, the origins of this enhancement [3-6] are believed to have contributions from both electromagnetic enhancement (EM) and chemical enhancement mechanisms. The latter involves charge transfer (CT) excitation [4-6] between detecting molecules and metal particles, whereas the former originates from a resonance between the incidence and scattered radiation fields associated to the excitation of localized surface plasmon resonances (LSPR) [7], termed as 'hot-spots’ [2,7,8] around nanoscale metal particles or artificial architectures. The sensitivity and reproducibility [9-11] of SERS signal strongly relies on different fabricated hot-spots, in which a vital role is played by a SERS substrate. In general, SERS substrate can be divided into two fundamental classes, random and artificial substrates [12]. Both of them should possess enough surface area to absorb more molecules to contribute to the Raman scattering and abundant hot-spots to enhance the local electromagnetic field. However, random substrate, such as colloidal, is proved to be limited because of weak reproducibility and fractal nanoparticle aggregation, leading their enhancement factors to decrease with increasing fractal size [2]. For the artificial nanostructure, the fourth power of local electromagnetic field of the hot-spots contributes to the signals of SERS and is sensitive to the critical dimension of artificial nanostructure [5,13]. To date, however, it is a challenge to control the nanostructures with extremely small size. Typically, previous engineering nanostructures were resorted to lithography-based nanotechnologies, involving electron-beam lithography (EBL), nanoimprint (NIL), nanosphere lithography (NSL), electrochemical lithography [14], and so on. For example, some arbitrary two-dimensional (2D) dimer nanostructures with small gaps such as bowties and nano-antennas, were proposed and prepared by EBL [15-25]. Some nanostructures were fabricated by NIL such as nanograting [26] and nanopost [27] as uniform SERS hot substrate. However, the major limitation lies in the sophistication of the fabrication processes and the inevitable defect.

Triangular noble nanoparticle arrays were fabricated by NSL [24,27]. Recently, nanocrescent [28,29] as a quasi-three-dimensional (3D) and tuning resonance SERS substrate was fabricated by NSL, which resorted by glancing angular metal deposition onto nanospheres. However, it is difficult to fabricate large-area and uniform 3D nanostructures with small gaps between adjacent patterns because lithography-based techniques are isotropic and the resolution is limited. Previous investigations depended on wet etching and electrochemical method, a typical example is pyramidal pits [30,31]; these engineering structures had large pitches which are much larger than the excitation laser probe spot size and lead to SERS enhancement with poor reproducibility and sensitivity. It is of crucial importance to develop 3D metal nanostructures with controllable nanogap sizes for the generation of strongly localized field. Van Duyne [32] and Fang [2] proposed metal films over nanosphere (MFON) electrodes as SERS active substrates in order to improve the surface nanostructure stability and suppress the inherent loss, where nanocavities with hot-spots are presented. However, the MFON structures are disposable substrates. Therefore, it is demanded to investigate reusable and high-sensitivity SERS substrates.

Here, we developed an NSL technique to produce large-area subwavelength 3D nanostructures performed as SERS substrates with high sensitivity, the SERS enhancement factor up to 10^11^, with high reproducibility, and especially free with adhesive layer. Hexagon-close-packed (hcp) 3D nanostructure arrays were fabricated with precise nanogaps. Three types of nanostructures were obtained by controlling etching parameters, involving hemispherical nanostructure (HS), hemi-ellipsoidal nanostructure (HE), and pyramidal pits. We proved the detrimental influences of the adhesion layer between noble metal layer and quartz substrate to the SERS enhancement. Such kind of SERS substrate is a reusable substrate which can be reused simply by removing and redepositing the metal thin film.

## Methods

### Monolayer of long-range-ordered polystyrene (PS) polystyrene as mask

Two hundred-nanometer monodispersed polystyrene (PS) nanospheres were synthesized by emulsifier-free emulsion polymerization, which would perform as colloidal mask of quartz substrate. The diameter of PS nanosphere was 200 nm with a standard deviation within 2 nm. A monolayer, long-range-ordered, large-area (more than 2 cm^2^), and hcp PS nanosphere was coated onto a cleaned quartz substrate by self-assembly. All quartz substrates were pre-treated with hydrophilic solution (H_2_O_2_/NH_3_^.^H_2_O/H_2_O 1:1:5 (*v*/*v*/*v*)) at 70°C for improving the stability of long-range-ordered nanosphere. The samples of surface-assembled PS nanospheres were baked on hotplate at 70°C for 5 min to remove some solvents.

### Assembly of detecting molecules

After etching the quartz substrates, all samples should be cleaned in butanone under ultrasonication for 2 min to remove organic residues and other particles. Consequently, a desirable noble metal (Ag, Au, Al, or Pt) thin film was directly deposited onto the surface by electron-beam evaporation. However, it was not necessary in the additional coated adhesive layer between the noble metal and quartz substrate, such as Cr or Ti. The samples with deposited metal thin film were soaked overnight in Rhodamine 6G (R6G)/methanol solutions. Two kinds of concentrations were used for nanopatterned samples and unpatterned for contrast samples, 10^-9^ and 10^-3^ mMol/L, respectively. The R6G-coated samples were rinsed three times in 10 mL of deionized (DI) water and blow-dried in nitrogen.

### Characterization

The top morphologies and the cross section of the samples were characterized by a FEI Sirion 200 field scanning electron microscope (SEM; Hillsboro, OR, USA) with acceleration voltages ranging from 5 to 10 kV. The SERS spectra were collected in backscattering mode by a JY LabRAM HR Raman spectrum (Horiba, Kyoto, Japan) with a laser wavelength of 633 nm. In order to achieve comparable Raman signal intensities on various samples with significantly different Raman signal enhancements, we fixed exciting laser with the pump power 0.6 mW and the integration time 20 s. In each sample, we measured 10 points to obtain average Raman intensity as the reference used in the SERS enhancement factor calculation. The Raman peaks fitted from the baseline-removed Raman spectra using a Guassian-Lorentzian lineshape.

### Calculation of SERS enhancement factors

We calculated the SERS enhancement factors of the single R6G molecule absorbed on our 3D nanostructures by the equation [3,4,12]

(1) $$\mathrm{EF}=\frac{I_{\mathrm{SERS}}/N_{\mathrm{surf}}}{I_{\mathrm{bulk}}/N_{\mathrm{bulk}}},$$

where EF was the enhancement factor, *I*_SERS_ and *I*_bulk_ are the Raman signal intensities at 1,365 cm^-1^ band, which is a characteristic representative vibration wave number of R6G molecules adsorbed on the 3D nanostructure and from the bulk R6G, respectively; *N*_surf_ and *N*_bulk_ are the numbers of the R6G molecules absorbed on the 3D nanostructures and the bulk R6G molecules exposed to the laser spot, respectively.

## Results and discussion

**Figure 1:**
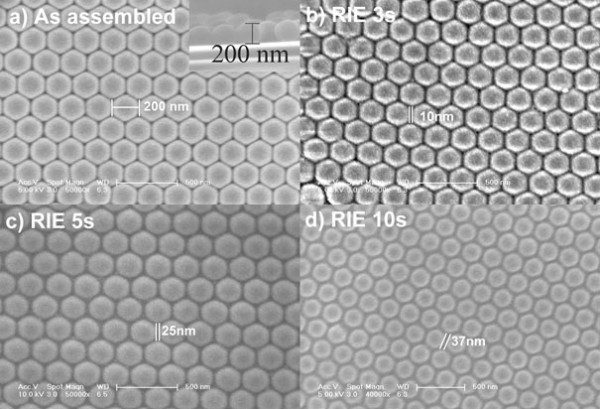
**SEM images of PS nanospheres on quartz substrate. (a)** Top morphology after self-assembly, and after O_2_ plasma tailoring with a typical gas pressure of 2 Pa, and O_2_ gas flow of 40 sccm, a radio-frequency (RF) power of 40 W, with the treatment time as **(b)** 3, **(c)** 5, and **(d)** 10 s, respectively.

The 3D nanostructural quartz substrates for SERS enhancement were fabricated by NSL. In detail, monolayer hcp-packed PS nanospheres were coated on the quartz substrate by self-assembly. Consequently, the PS-coated quartz substrate was precisely tailored by O_2_ plasma in a RIE system after removing solvents, using a recipe as radio frequency (RF) power of 40 W, pure O_2_ gas flow of 40 sccm, and chamber pressure of 2 Pa. We found that the lateral and vertical etching rates of PS nanosphere under this condition were both 300 nm/min. Such high lateral etching rate was suitable to tailor PS nanosphere, while for etching PS nanosphere, the O_2_ gas flow should be changed to 5 sccm so that the lateral etching rate can be lowered to 10 nm/min and the vertical etching rate as 30 nm/min. Figure 1 illustrates the results after tailoring PS nanosphere under above recipe with different operating time. Figure 1a is a typical SEM image of hcp-packed PS nanosphere without tailoring and the inset picture is its cross-sectional SEM image; both of them demonstrated that the sample was a monolayer PS nanosphere dispersed on quartz substrate. With the O_2_ plasma operating time increased from 3 to 5 and 10 s, and the gaps between two adjacent nanospheres were increased from 10 nm to 25 and 37 nm, as shown in Figure 1b,c,d, respectively. Figure 1d also illustrates substantially that the top morphologies were bleary after 10-s RIE treatment. Since PS nanosphere contacted with the quartz substrate only at one point, the whole sphere was etched through gaps. The geometry of the etched nanosphere is a crucial factor for the followed substrate etching to achieve 3D nanostructures.

The quartz substrate was directly nanopatterned by RIE. The tailored PS nanosphere performed as the sacrificial mask. The final geometries, sizes, and nanogaps between two adjacent architectures were changed with the mixture of the etching gases, involving CF_4_/CHF_3_/SF_6_/Ar/O_2_. We first characterized the etching rate of PS nanosphere and quartz substrate under each individual pure etching gas (CF_4_/CHF_3_/SF_6_/Ar/O_2_) at a RF power of 40 W and a typical gas pressure of 2 Pa. And then according to the etching results of the above individual gases, we designed several reasonable etching recipes with the mixture of the above gases. It was found that the scale of PS nanosphere was gradually reduced, and therefore, the gap of two adjacent nanospheres was also gradually increased. The quartz substrate was nanopatterned and kept the same, gradually changing with the gradual change of PS nanosphere mask.

**Figure 2:**
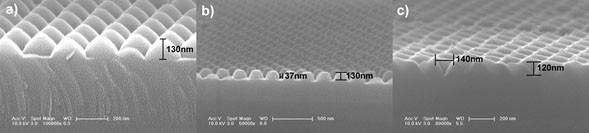
**SEM images of nanopatterned quartz substrates with different nanoscale geometries after direct RIE etching with CF_4_/CHF_3_/SF_6_/Ar/O_2_ mixing gases. (a)** hemisphere nanostructure, **(b)** hemi-ellipsis nanostructure, and **(c)** pyramidal pit nanostructure.

To achieving different 3D nanopatterned quartz substrate, the vertical and lateral etching rate should be extremely controlled by varying the ratio of gas components. As for the hemisphere geometry, the ratio of the lateral and vertical etching rate should be precisely controlled and ranged from 1 to 1.2 with the composition and gas flow of the etching gases as CF_4_ (26 sccm)/CHF_3_ (10 sccm)/SF_6_ (24 sccm)/Ar (5 sccm)/O_2_ (10 sccm). For the ellipsis geometry, the ratio should range from 1.4 to 1.8 with the composition and gas flow of the etching gases as CF_4_ (26 sccm)/CHF_3_ (5 sccm)/SF_6_ (40 sccm)/Ar (5 sccm)/O_2_ (5 sccm), whereas for the pyramidal pits geometry, the ratio should range from 2 to 2.5 with the composition and gas flow of the etching gases as CF_4_ (20 sccm)/SF_6_ (40 sccm)/Ar (5 sccm)/O_2_ (5 sccm), respectively. Figure 2 shows the results by direct RIE etching with above-discussed mixing gases. Figure 2a illustrates the SEM image of patterned quartz substrate with hemisphere geometry, whose structural parameters are the diameter of 200 nm, the height of sphere coronal of 130 nm, and the nanogaps between two adjacent architectures below 5 nm. It seems that the two adjacent engineered architectures are tangential, with a point contact. Except the points of tangency, the top morphology was a gradually changed curve. Figure 2b presents a hemi-ellipsis geometry, with structural parameters as sub-axle of 200 nm and height of 130 nm. Figure 2c shows the pyramidal pits with structural parameters as opening of 140 nm and depth of 120 nm. The gap was defined as the distance between the edges of two adjacent architectures on top surface. The side surface of this engineered architecture was flat. So far, much effort to fabricate pyramidal pit geometry was based on wet etching technique-induced large engineered architectures which limited their potential application [30,31]. Here, we successfully fabricated three different engineered 3D nanostructures with large-area, long-ordered, and controlled morphology by direct dry etching process and NSL technique.

Above fabrication procedures, providing a simple and spatially controllable method on the nanoscale structures according to rational etching parameters, are instrumental in developing SERS substrates. The motivations for the 3D noble metallic nanostructural substrates are to create large-surface area and high-surface dense hot-spots to contribute to SERS with a large enhancement factor, to improve enhancement reproducibility, and to resolve the problem of adhesion layer. The 3D nanostructures would cause the incident light to converge, amplify the total absorption of excitation light and increase the effective cross section of Raman scattering. The geometries, sizes, and gaps of these 3D nanostructures all affect the surface plasmons (SPs). In this article, SERS spectra were collected at 633-nm laser wavelength. The R6G molecules were employed as detection target. Before the R6G molecules were dosed onto the nanostructures, a desirable noble metal (Ag or Au) was directly deposited onto the surface by electron-beam evaporation on the fabricated three types of 3D nanostructures and unpatterned substrate, and then the samples were soaked overnight in R6G/methanol solutions. Two kinds of bulk concentrations were used for nanopatterned samples and unpatterned for contrast samples, 10^-9^ and 10^-3^ mM, respectively. The R6G coated samples were rinsed several times in 10 mL of DI water and blow-dried in nitrogen.

**Figure 3:**
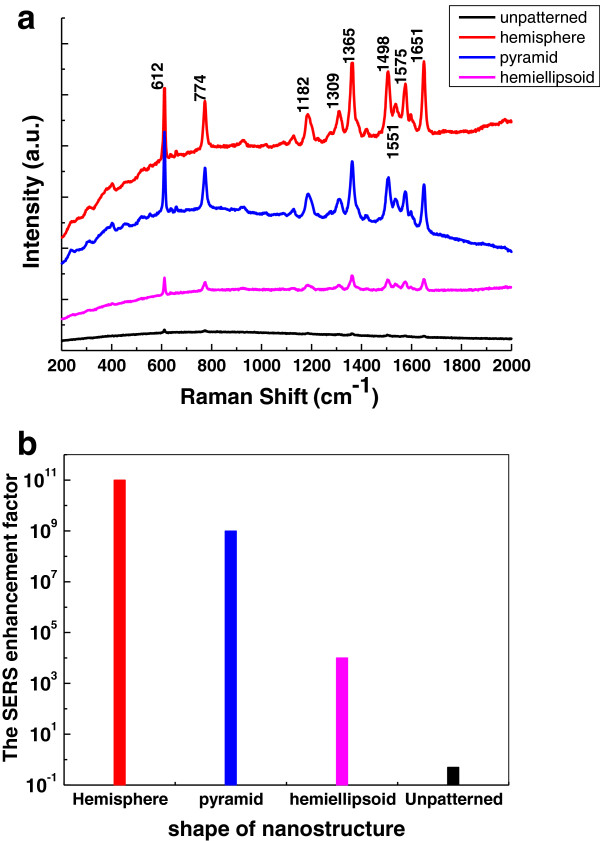
**SERS spectra of monolayer R6G (a) and average SERS enhancement factor EF (b). (a)** Monolayer R6G is absorbed on three types of 3D Ag nanostructures, with laser power 1.8 mW and the integration time 10 s. The SERS spectrum of the unpatterned Ag film was amplified 40-fold and performed with laser power 9 mW, the integration time 20 s, and the concentration of R6G 10^-3^ mM. **(b)** Average SERS enhancement factor EF as the function of the geometries.

The influences of geometries, nanogaps, and adhesive layers of these 3D nanostructures on the Raman scattering enhancement were quantified. The SERS enhancement factors of hemispherical, hemi-ellipsoidal, and pyramidal pits were about 10^11^, 10^6^, and 10^8^, respectively. Figure 3 shows the SERS spectra of R6G monolayer molecules absorbed on the Ag film which was deposited on unpatterned (black curve) and three types of 3D nanostructure substrate, separately. The SERS signal of the unpatterned film was collected at the laser power of 0.6 mW and the integration time of 20 s. The signal was amplified 40-fold; all peaks were very weak. The red, blue, and magenta curves were the SERS signals of the hemispherical and pyramidal pits and hemiellipsoid nanostructures, respectively, which were collected at the integration time of 10 s. The SERS intensity of hemispherical nanostructure was the strongest. For this SERS scattering detection, the structural parameters were fixed with 200-nm pitch and 130-nm height. The SERS enhancement factor of hemispherical nanostructure achieved 10^11^. Three factors contributed to the strong SERS intensity: active area, narrow nanogaps, and cross-sectional area [4,5]. First, the large area and long-range-ordered nanostructure increased the SERS effect; therefore, the density of hot-spots were enormous in the Raman scattering volume and increased the average SERS intensity. Second, the Raman scattering spectra of the nanostructures was a function of the localized surface plasmon resonance, and the interaction between the localized surface plasmon (LSP) and the Raman scattering light further enhanced the local electromagnetic fields. Both of them depended on the narrow nanogap distribution. Third, the gradual hemispherical nanostructures could enhance the Raman cross-sectional area by amplifying the incidence signal of the radiation and absorption. Although, the hemiellipsoidal structural parameters were kept the same with the hemispherical nanostructure, starting from the PS diameter as 200 nm, etching depth as 130 nm, and all deposited with 20-nm Ag film. The SERS average enhancement factor of hemiellipsoidal nanostructure was only about 10^6^, smaller than the hemispherical nanostructure. Among these three structures, the distance between two adjacent hemiellipsoidal structures was the largest. The SERS enhancement factor of pyramidal pits was about 10^8^, which was smaller than the hemispherical nanostructure; however, larger than the hemiellipsoidal nanostructure, and also larger than the previous literatures [30,31]. Although the three sharp vertices of the surface grids and bottom points of pyramidal pits constructed the hot-spots, the scale of top-surface triangular grid of the pyramidal pits was still small enough to concentrate the light and boost the SERS enhancement. The tunable SERS signals altered with the controllable nanogaps (Additional file 1: Figure S1). Such kind of SERS substrate is a reusable substrate which can be reused simply by removing and redepositing the metal thin film (Additional file 1: Figure S2).

**Figure 4:**
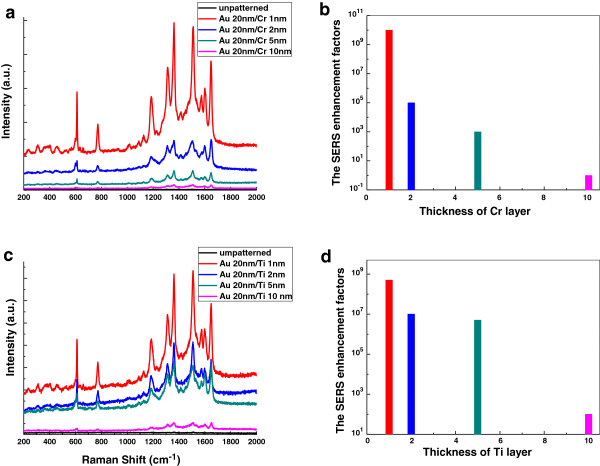
**SERS spectra (a,c) and enhancement factor (EF) of monolayer R6G adsorbed on hemispherical nanostructures (b,d).** Nanostructures with different thicknesses of adhesion layer. **(a,b)** Cr (Chromium). **(c,d)** Ti (Titanium) between the quartz substrate and noble metal film. The unpatterned samples were coated with 5-nm-thick adhesive layer.

Almost every experimental study of SERS omitted the issues of the negative effects of adhesion layer [32-36], while we found that it had a dramatic influence of SERS enhancement. Since noble metals possess (involving Au, Ag, Pt, and so on) poor adhering ability to quartz substrate, an artificial adhesion-promoting intermediate layer between noble metal and quartz substrate, such as Cr (Chromium) or Ti (Titanium) is needed. However, the intermediate layer Cr or Ti would greatly shift and broaden the surface plasmon resonance. The magnitude of resonance damping has also been found when the thickness of the adhesion layer increases. Fortunately, our 3D nanostructures could resolve the adhesion-promoting intermediate layer issue because the noble metal deposition procedure was the final step, which avoided influence on the chemical reagents and poor adhering ability. To extensively compare the results between with and without intermediate adhesion layer, we executed the experiments with the different thickness of adhesion layer and employed the hemispherical nanostructure coated with a 20-nm Au film, with fixed structural parameters and other conditions. Figure 4a,b illustrates the negative influences of Cr and its foundation of the SERS enhancement factors. It was found that the detrimental contribution to the Raman signals and the SERS enhancement were significantly attenuated with increasing several nanoscale thickness of the Cr adhesive layer. When with the 1-nm Cr layer, the average SERS enhancement factor was about 10^10^. With the 2-nm Cr layer, the SERS enhancement factor was declined to 10^5^, with 5 nm, down to 10^3^. While with the 10-nm Cr layer, the Raman signals were so weak that some fingerprint peaks of R6G molecule was disappeared, similar with the result of the unpatterned Au 20-nm film sample on quartz substrate. Ti, as the adhesive layer, possessed the similar tendency. While different with Cr adhesive layer, the detrimental influence to the Raman signals generated by 2- and 5-nm-thick Ti was almost the same. Their average SERS enhancement factors were about 10^7^. With the 10-nm Ti adhesive layer, the fingerprint peaks of R6G molecule also downed near zero. The SERS enhancement factors were below 10^2^. There were no Raman signals from the unpatterned sample when deposited with 5-nm adhesion-promoting Cr or Ti layer between quartz substrate and 20-nm Au layer (the black curves of unpatterned sample shown in Figure 4a,c). In order to minimize the detrimental influences of adhesion layer and still can identify molecular species, our experiments provided a persuasive evidence that thinner adhesive layer was more favorable to the SERS enhancement factor, we suggested that an appropriate thickness of the Ti adhesive layer below 5 nm; however, Cr should be used below 2 nm. We believed that a strong damping of plasmonic resonance due to increased absorption in the adhesive layer. The negative effect of losses was confirmed by the low enhancement for Cr compared with Ti, the absorption of Cr was about three times of Ti at the wavelength of a 633-nm laser, and by the fact that the Raman enhancement increased when the adhesion layer thickness decreased. Lastly, the damping effect of absorption was also exhibited for dielectrics, with a higher enhancement for Ti than for Cr.

## Conclusions

We addressed a prompting nanosphere lithography method for fabricating spatially controllable 3D nanostructures, and successfully achieved hemisphere, hemiellipsoid, and pyramidal pit-shaped nanostructures. We demonstrated that the kernel factor was to precisely control the ratio of the lateral and vertical etching rate to achieve the desirable geometries. Effective and extreme tailoring of the diameter of the PS nanosphere mask played a crucial role in achieving the controllable nanogaps between these nanostructures, which could be below 10 nm or even at point contact between two adjacent nanostructures. Applying the reliable 3D nanostructures as tunable SERS substrates, we extensively study influences of geometries, nanogaps, and the adhesion layer between the desirable noble metal and the underlying quartz substrate on SERS enhancement effect. Negative contribution of adhesive layer was demonstrated according to the results of SERS enhancement factors. The tunable SERS substrates possess great advantages: (1) achieving strong average SERS enhancement factor up to 10^11^; (2) free-adhesion layer; (3) a platform for any desirable metal, and can be reused by simply removing and redepositing the metal film while not destructing the 3D nanostructures or repeating the tedious fabricating procedures. Due to the increase in damping plasmonic resonance with increasing the thickness of the adhesion, we suggest the suitable adhesion of Ti layer below 5 nm and of Cr below 2 nm.

## Competing interests

The authors declare that they have no competing interests.

## Authors’ contributions

ZZD and QQL conceived and designed the experimental strategy. ZZD prepared and performed the experiments and wrote the manuscript. QQL and BBF helped with the editing of the paper. All authors read and approved the final manuscript.

## Supplementary Material

Additional file 1 Influence of nanogaps in the 3D nanostructures and reusability of the SERS substrate. Click here for file
