# Supplementary material for: Reusable three-dimensional nanostructured substrates for surface-enhanced Raman scattering
Source: Nanoscale Res Lett. 2014 Jan 13;9(1):25. doi: 10.1186/1556-276X-9-25 (PMC3898998; doi:10.1186/1556-276X-9-25)
Supplement: Additional file 1 — Influence of nanogaps in the 3D nanostructures and reusability of the SERS substrate. [file 1556-276X-9-25-S1.docx]

Supporting materials:

Reusable Three-Dimensional Nanostructured Substrates for Surface-Enhanced Raman Scattering

Zhendong Zhu^1,3^, Qunqing Li^1,2^*,Benfeng Bai^3^, Shoushan Fan^1,2^

^1^State Key Laboratory of Low-Dimensional Quantum Physics, Department of Physics & Tsinghua-Foxconn Nanotechnology Research Center, Tsinghua University, Beijing 100084, China

^2^Collaborative Innovation Center of Quantum Matter, Beijing, China

^3^Department of Precision Instruments, State Key Laboratory of Precision Measurement Technology and Instruments, Tsinghua University, Beijing, China.

*Corresponding author. Tel: +86 10 62796019; Fax: +86 10 62792457.

E-mail address: [QunqLi@mail.tsinghua.edu.cn](mailto:QunqLi@mail.tsinghua.edu.cn)

Influence of nanogaps

The hemispherical nanostructure was utilized as SERS substrate and the nanogap between two adjacent hemispherical nanostructures was changed from below 5 nm to 60 nm, without altering other parameters or conditions. The Raman intensity variations and the tendency of the SERS enhancement factors of R6G molecule on the 3D substrate with various nanogaps were illustrated in Fig.S1a and S1b, respectively.

**Figure S1.** (a). The SERS spectra and (b) SERS enhancement factor EF of monolayer R6G absorbed on the 3D SERS substrate with different nanogaps between two adjacent hemispherical nanostructures.

Reusability of the SERS substrate

The hemispherical nanostructure was utilized as SERS substrate and the reusability was studied by depositing a new gold film after removing the previous gold film along with detected molecule by wet-etching method, without altering other parameters or conditions. Figure 2S give the results of the Raman intensity variations of R6G molecule on such SERS substrate. The Raman intensity of the detecting molecule does not change with the re-used SERS substrate. The SERS enhancement factor also achieves up to 10^11^, only with a small change. It gives an evidence that the sensitivity of our SERS substrate depends on the geometry of the nanostructure, which has been confined into quartz substrate. The results demonstrate that our SERS active substrate is reusable.

**Figure S2**. The SERS spectra of monolayer R6G absorbed on the pristine deposited SERS substrate and re-used SERS substrate.

Figure S1 Z. Zhu et al
